# Barriers and facilitators to sports participation in autistic Europeans: insights from a large-scale questionnaire survey

**DOI:** 10.3389/fspor.2025.1580462

**Published:** 2025-07-16

**Authors:** Célia Ruffino, Nicolas Gueugneau, Sidney Grosprêtre

**Affiliations:** ^1^ Laboratory C3S, Culture Sport Health and Society, Sport and Performance Department, University of Franche-Comté, Besançon, France; ^2^ Institut Universitaire de France (IUF), Paris, France

**Keywords:** autism, Europe, sport practice, physical activity, barriers to participation, questionnaire-based research

## Abstract

**Background:**

Autism is a neurodevelopmental condition affecting both social interactions and individual motor coordination with a wide spectrum of characteristics and support needs varying significantly across individuals. Given the increasing prevalence of autism, effective interventions are crucial to improving quality of life. Physical activity has been recognized as a valuable tool for enhancing physical fitness and reducing autism-related traits, such as repetitive behaviors and social difficulties. However, autistic individuals tend to be less active than non-autistic. The SACREE Sport & Autism project, part of the European ERASMUS + initiative, aims to bridge the gap between standard sports recommendations and autistic individuals’ needs. This study seeks to provide an overview of sports participation among autistic Europeans and identify factors influencing their engagement in physical activities.

**Method:**

Using an online questionnaire translated into five languages, data was collected from 540 respondents across several European countries. Most responses were provided by parents or caregivers (64.3%), while 25.5% came directly from autistic individuals.

**Results:**

Findings revealed that 71.2% of respondents engage in regular physical activity, with an average of 2.45 sessions per week lasting approximately 65 min each. Individual sports dominate, comprising 79% of reported activities, with aquatic exercises being the most common. While many participants acknowledge the benefits of physical activity, 74% believe that sports are not sufficiently accessible for autistic individuals. The primary reasons for non-participation include a lack of suitable facilities (54.1%) and uncertainty about where to practice (22.2%).

**Conclusions:**

The study underscores the importance of structured physical activity in improving both physical health and autism-specific characteristics. However, the findings highlight discrepancies between current practices and recommended physical activity levels. Increased accessibility to adapted sports programs, better awareness campaigns, and policy reforms are needed to encourage greater participation. Furthermore, the study suggests that practice type plays a key role, with individual, predictable activities being preferred over dynamic, team-based sports. By shedding light on sports habits among autistic individuals, this research provides a foundation for tailored interventions and public policies aimed at fostering an inclusive and sustainable sports culture across Europe.

## Background

Autism encompasses a broad range of neuropsychological conditions, characterized by differences in social interaction and communication, as well as potential impacts on sensory processing and motor coordination. Socially, autistic people often experience, for instance, difficulties in understanding others’ behavior or interpreting social and emotional cues (1). At the individual level, they may exhibit impairments in motor coordination (2) or engage repetitive and stereotypical movements (3); which could significantly alter quality of life (4). It is therefore essential to propose various interventions aiming at improving the quality of life of autistic people, especially as prevalence rates continue to rise. Indeed, a recent review highlighted a general increase in prevalence, which has been attributed to several factors such as greater community awareness and advancements in case identification and diagnostic criteria (5). More precisely, in Europe, the average prevalence was 80 per 10,000 individuals between 2000 and 2020 (6), highlighting its status as a major public health concern.

It is well-established that individuals with autism can benefit from therapeutic interventions designed to reduce or eliminate these maladaptive behaviors (7). Since the late 20th century (8), many research has focused on the potential benefits of physical activity, not only for improving physical functions (9, 10) but also for reducing specific characteristics associated with autism (11, 12). Indeed, engaging in individual (13–15) or team sports (16–18) is correlated with a reduction in the severity of autism characteristics, such as stereotyped behavior or difficulties in communication and social interaction. After three months of regular practice (three times per week of 45 min Tai Chi Chuan training sessions), a significant decrease up to 25% of composite autism scores, measured by the recognized Gilliam Autism Rating Scale 2 Score (19), can be observed (20). This included decreases in behaviors such as avoiding stable eye contact and wing hand movement.

However, it appears that autistic individuals are generally less physically active than their non-autistic peers (21–24), which may negatively impact various functions, including bimanual coordination. Indeed, as shown in the study by Norouzi et al. (25) inactive autistic children exhibited decreased mu rhythm suppression and poorer performance in bimanual coordination tasks compared to their active counterparts, suggesting that physical activity may play a key role in enhancing both motor skills and associated neural processes in autism. This reduced level of physical activity for autistic individuals may be partly explained by the general impairments that affect their daily functioning (26), as well as by limited accessibility and a lack of awareness regarding the importance of engaging in regular physical activity. Indeed, key determinants influencing the effectiveness of interventions include factors such as the type of training and, importantly, the dose-response relationship. According to the World Health Organization (WHO), it is recommended to practice at least 150 min of moderate-intensity or 75 min of vigorous-intensity physical activity per week.

In this context, our study is part of an ERASMUS + project, a flagship program of the European Union aiming at promoting cooperation and mobility in education, training, youth, and sports. The project SACREE *Sport & Autism* is dedicated to fulfil the gap between usual sport recommendations and autistic individuals. This initiative addresses a dual objective: on the one hand, to provide a comprehensive overview of Autistic Europeans’ sports practices and, on the other hand, to identify the determinants that encourage or hinder regular physical activity. This study employs a quantitative approach based on an online questionnaire administered to a diverse population from several EU member states. It seems to be crucial, through this participative research including autistic community (27), to understand the motivations, barriers, and behaviors associated with sports participation, to designing and promoting effective and appropriate public policies.

The objectives of this study are threefold. Firstly, we examined the frequency and types of sports activities practiced by European citizens, as well as the available infrastructures and resources. Secondly, we explored the individual and contextual factors influencing sports participation, including age, gender, environment and motivations. Thirdly, we aimed to identify perceived barriers and potential levers to promote greater participation in physical and sports activities.

The results of this research contribute significantly to the existing literature on sports sociology and public health. Moreover, they will provide a solid foundation for developing policy recommendations and concrete initiatives to promote a sustainable sports culture in Europe. By integrating comparative perspectives between countries and highlighting best practices, this study aims to foster constructive dialogue among institutional, associative, and academic stakeholders involved in sports development across Europe.

## Methods

The choice of an online questionnaire as the primary data collection tool is based on several considerations. First, this method enables access to a large sample of participants across different European territories while reducing the costs and logistical constraints associated with field surveys. Second, the anonymity provided by this mode of response fosters honest and spontaneous participant feedback, thereby enhancing the reliability of the responses. Finally, the standardization of questions ensures comparability of results across countries and age groups.

**Figure 1:**
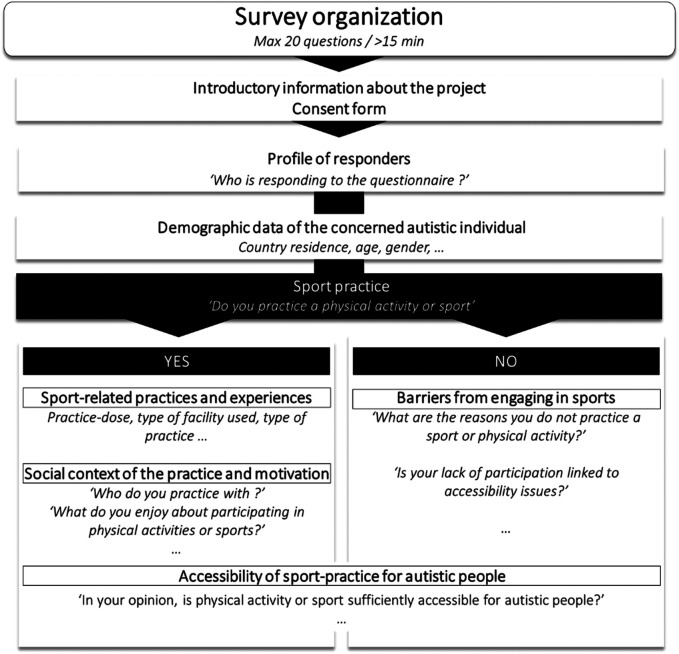
General organization of the survey. y.o., years old.

Thus, an online survey was disseminated by the European partners of the project through their respective regional and national networks in the field of Autism and sport, and was translated into five different languages: English, French, Italian, Portuguese, and Croatian (Figure 1). Participants were primarily recruited through convenience sampling, based on their accessibility and willingness to participate. Additionally, a snowball sampling approach was employed, where initial participants referred other potential participants from their networks, particularly to reach individuals who might otherwise be difficult to access.

At the beginning of the survey, participants were provided with detailed information about the purpose and scope of the European project. They were also given the opportunity to electronically sign the consent form, thereby providing their informed consent for data collection. Notably, the introductory information specified that the survey could be completed by a third party on behalf of the autistic individual, such as a parent, sibling, caregiver, or another representative. One of the first pieces of information collected in the survey concerned the profile of the respondent—whether it was the autistic individual themselves or a relative or caregiver. Regardless of who completed the survey, all questions were designed to assess the opinions and experiences of the autistic individual.

Importantly, no personal data, such as names, addresses, or phone numbers, was collected to ensure anonymity and data privacy. However, participants were given the option to voluntarily provide their email addresses if they wished to stay informed about the progress and outcomes of the project.

The survey aimed to gather information across a wide range of areas. Initially, demographic data such as the participant's country of residence, age, and gender were collected. Following this, the survey was divided into two main sections based on the participant's response to the initial question regarding sport and physical activity: “Do you practice a physical activity or sport?”

If the participant answered “yes”, they were directed to a dedicated section focusing on their sport-related practices and experiences. This section included questions about the specifics of their physical activity or sport practice, such as the number of sessions per week, the average duration of each session, and the type of facility used (e.g., professional club, home, or other settings). Additionally, it explored the social context of their practice, such as whether they engaged in activities with other autistic individuals, family members, or others. This section also delved into the motivations behind engaging in sport, with questions like, “What motivated you to start practicing a sport?” and “What do you enjoy about participating in physical activities or sports?”. Furthermore, participants were asked for their opinions on broader topics related to sport and autism, including accessibility, with questions such as, “In your opinion, is physical activity or sport sufficiently accessible for autistic people?”.

For participants who answered “no” to the initial question about practicing physical activity or sport, a separate, shorter section was presented. This section aimed to understand the barriers that prevent individuals from engaging in sports or physical activities. Questions included, “What are the reasons you do not practice a sport or physical activity?” and “Is your lack of participation linked to accessibility issues?”. Similar to the previous section, participants were asked for their perspectives on general topics related to sport and autism, including the accessibility of sports for autistic individuals.

The survey was designed to be concise and user-friendly, containing no more than 20 questions, depending on the participant's responses and the section they completed. It was calibrated to take no more than 15 min to complete. To ensure accessibility and ease of use, all questions were presented in a multiple-choice format, with no open-ended questions included.

Data are presented in percentages and mean ± SD for the following variables: participant characteristics such as age and gender distribution, as well as for physical activity parameters including frequency of practice (number of sessions per week) and session duration (adjusted according to age). Descriptive statistics were performed using Microsoft EXCEL software. Other statistical analyses were performed using JASP Software [version 0.19, JASP 290 Team (2020), University of Amsterdam]. Two-tailed *t*-tests were performed for pairwise comparisons (e.g., males vs. females), while one-way ANOVAs were conducted to analyze differences in sport practice variables — specifically frequency and duration of sessions — across multiple groups such as different age categories. In case of a significant effect, a *post hoc* test with Bonferroni correction was performed. Statistical significance was set at *P* < 0.05.

## Results

### Profile of responders and demographic data

A total of 540 responses were gathered, from Portugal (*n* = 86), Italy (*n* = 173), Croatia (*n* = 68), France (*n* = 185) and various other European countries (*n* = 28). Of these, 64.3% were completed by siblings or parents of autistic individuals, 25.5% by autistic individuals themselves, and 10.2% by other caregivers.

**Figure 2:**
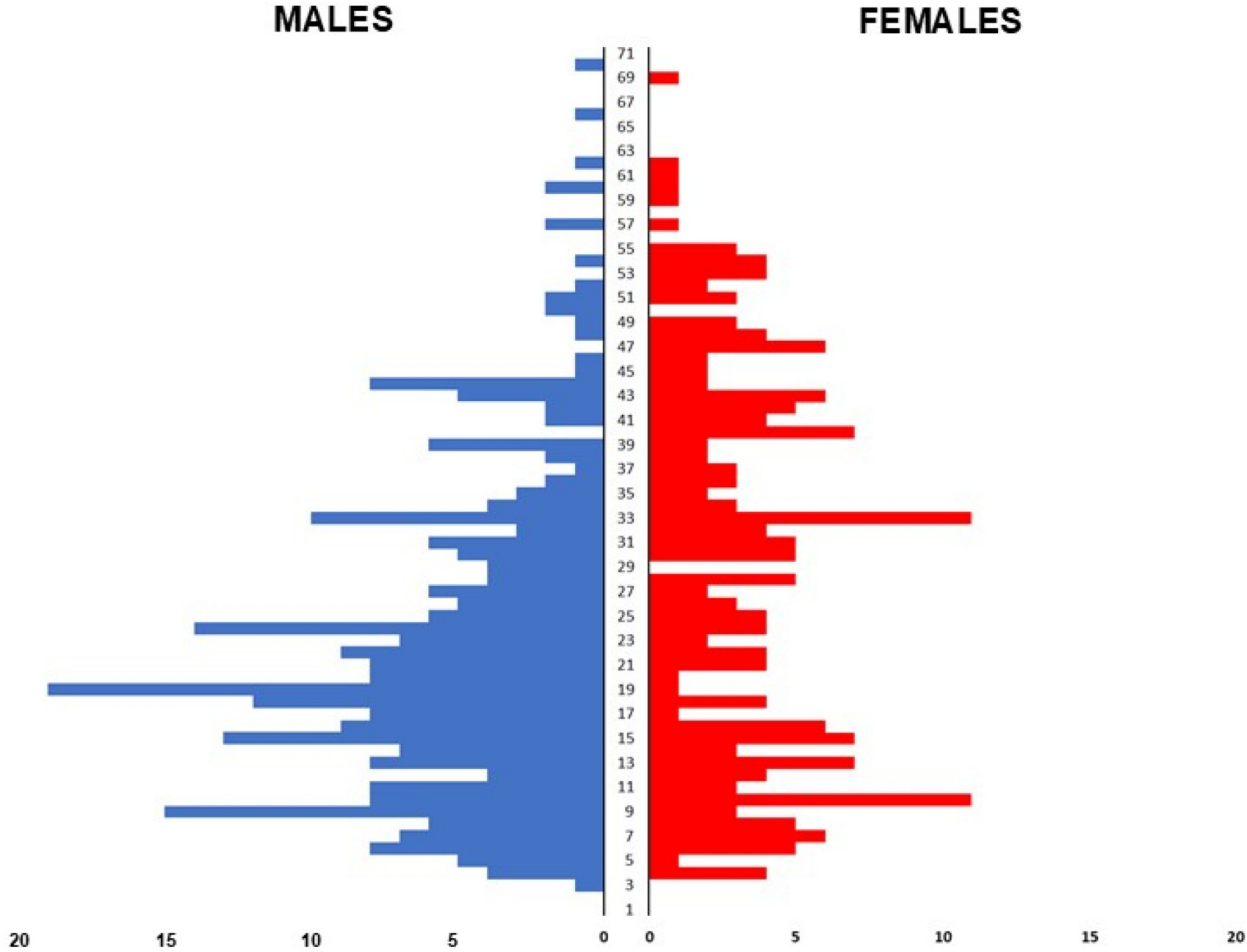
Age pyramid of the population.

The total population represented 57.7% of men (age: 23.0 ± 13.2 years old), 39.6% of women (age: 29.0 ± 15.8 years old) and 3.1% of non-binary persons (age: 27.7 ± 10.5 years old). The age distribution ranged from 3 to 71 years old among autistic individuals (total mean age: 25.6 ± 14.5 years old) (see Figure 2).

### Sport practice

Regarding sports and physical activity, 71.2% of the respondents reported engaging in regular physical activity, while 28.8% indicated they did not participate in any. The sport practitioners had a mean aged of 26 ± 14 years and composed by 59% males, 38% female and 3% non-binary individuals. As for the non-sport practitioners, their mean age was 25 ± 15 years, with 50% identify as females, 45% as males and 4% as non-binary.

### Sport practitioners

**Figure 3:**
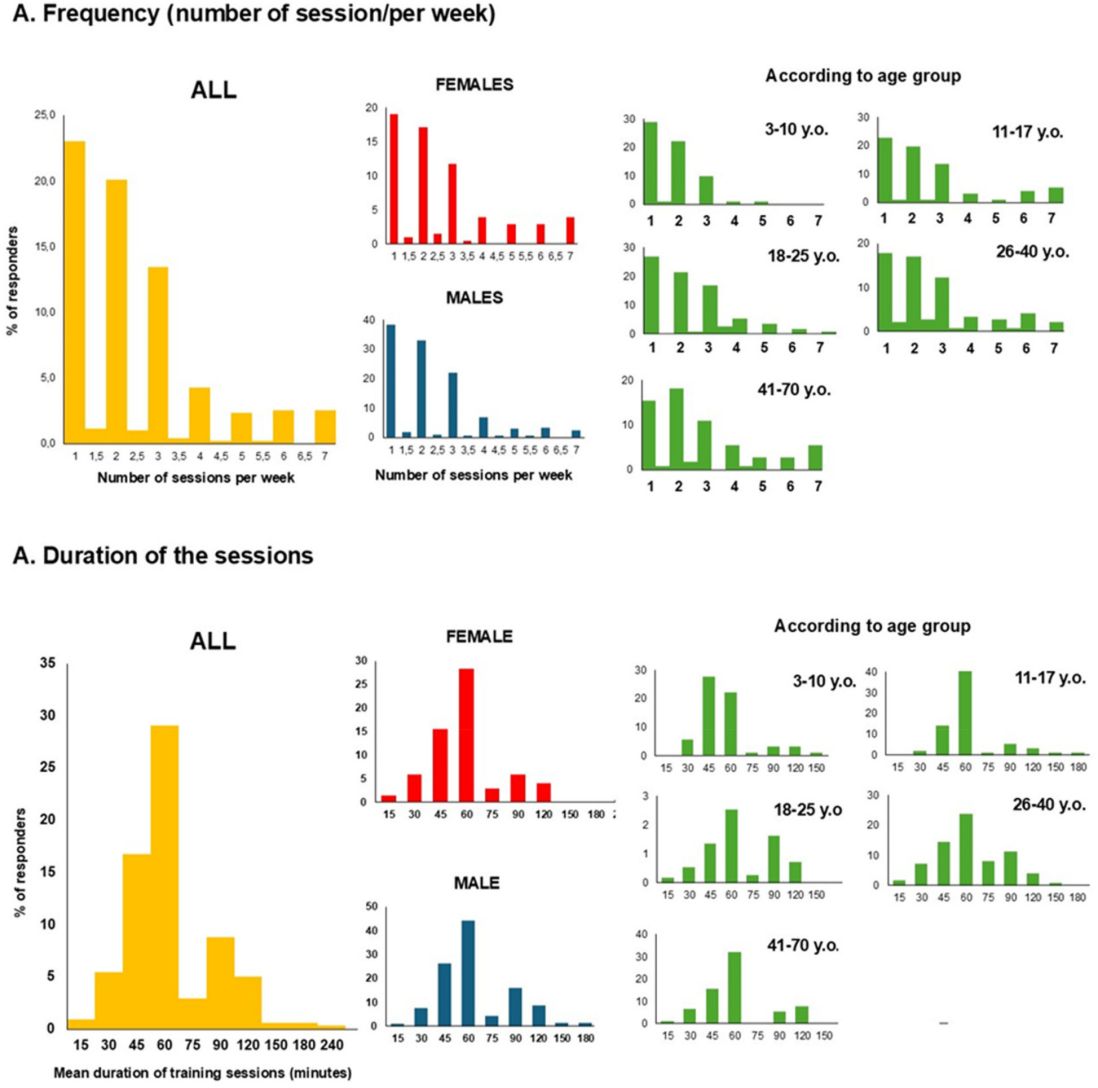
Sport dose of the population. y.o., years old.

Among those who practiced sports, respondents provided information on the practice-dose, including frequency and duration of their activities (Figure 3). The number of sports sessions per week ranged from 1 to 7, with a mean of 2.45 ± 1.6 sessions. The average duration of a training session was 65.7 ± 31.9 min, corresponding to a mean total of 115.22 ± 154.9 min of physical activity per week. Additionally, no significant differences were observed between men and women (*P* > 0.34). However, a main effect of age has been found on training frequency (F_4,386_ = 4.487, *P* = 0.001). Older age groups engaged in a higher number of sessions per week compared to younger groups. For instance, the average frequency for group age 3–10 years old was 1.8 ± 0.9 sessions per week, while group 41–70 years old was 2.8 ± 1.8 (*P* = 0.001). Similarly, a main effect of age group has been found on session durations (F_4,372_ = 3.300, *P* = 0.011). Session duration was shorter for children (age 3–10 vs. age 41–70: *P* = 0.006).

Concerning the context of their sports practice, 56.8% of respondents reported practicing in a sports club, 38.5% engaged in free practice, and 10.8% participated in a specialized structure. Additionally, 4.1% declared practicing exclusively in a school setting. 34% declared practicing their activity also in a competitive way, while 66% declared not practicing competition at all. We also asked the participants about their reasons for engaging in sports. Among practitioners, 37.8% reported being introduced to sports through their family, 33.1% started on their own, 12.2% were influenced by a friend, and 11.5% began through school sports.

Regarding the type of practice, respondents reported engaging in various sports activities (Table 1), with aquatic activities being the most common, practiced by 21.70% of participants. When distinguishing between individual or team sports, we observed that only 21% of the total practice involved team sports practice, such as soccer or basketball, while 79% consisted of individual sports. Among these individual sports, 9% were practiced in opposition, such as martial art or tennis. Finally, during the data analysis phase, we distinguished the nature of activities based on their practice characteristics, distinguishing between two opposites forms: open-skill sports, such as track and fields or swimming, performed in a static environment with standardized and predictable conditions, and closed-skill sports, such as racket or team sports, which are performed in dynamic situations where practice conditions are highly variable and unpredictable. The results revealed that only 37% of practice involved open-skilled sports, compared to 63% in closed-skill sports.

**Table 1:** Type of sport practiced.

Sport	Raw	%
Swimming	102	21.70%
Fitness	35	7.45%
Various	34	7.23%
Soccer	32	6.81%
Walking	30	6.38%
Biking	22	4.68%
Martial art	22	4.68%
Climbing	16	3.40%
Running	15	3.19%
Horse riding	12	2.55%
Rugby	12	2.55%
Track and field	12	2.55%
Basket-ball	11	2.34%
Danse	11	2.34%
Yoga	11	2.34%
Others	9	1.91%
Tennis	8	1.70%
Fencing	6	1.28%
Table tennis	6	1.28%
Golf	5	1.06%
Handball	5	1.06%
Volleyball	5	1.06%
Archery	4	0.85%
Badminton	4	0.85%
Boxing	4	0.85%
Surf	3	0.64%
Parkour	2	0.43%

Next, we questioned practitioners about the social context of their practice and their motivation. Among respondents, 37.2% reported practicing alone, 34.5% practiced with both autistic and non-autistic partners, 21.6% practiced exclusively with relatives (parents or siblings), and 10.8% declared practicing only with other autistic individuals. Concerning the choice of their practice, 55.4% declared practicing because they like this activity, 21.6% declared that they didn't have other choice (only activity available for them, etc.), 19.6% because it was the most practical (the closest structure, etc), and 12.2% because some relatives (friends, family) were practicing it.

Finally, when asked the practitioners about the accessibility of sport-practice for autistic people, only 27% stated it is accessible, while 74% responded that is not.

### Non-sport practitioners

Over the responder who reported not to practice any sport activity, 54.1% declared that the reason of not practicing was related to the lack of structure which accept autistic individuals. 22.2% declared not knowing where to practice. 6.17% declared not being attracted by sport practice, 3.4% not to have time for practicing sport, and the remaining respondents cited various other reasons (e.g., cost, distance, difficulty, etc.).

When asked if they think sport is accessible enough for autistic people, 35.5% answered “yes”, while 64.5% responded “no”.

## Discussion

In the context of a European ERASMUS + project, the SACREE Sport & Autism project aims to provide a comprehensive overview of sports practices among autistic Europeans and identify the factors that promote or hinder regular physical activity. To achieve this, we designed an online questionnaire to be completed by autistic individuals, their families or by their caregivers. With more than 500 answers, we gathered valuable insights into the sport practices of the European autistic individuals, including the frequency and the duration, the type and the context of their practice. This study also provides insight into the reasons why autistic individuals do not engage in sports.

### Practice dosage: a key approach to promoting health and reducing specific characteristics associated with autism

It is well known that engaging in sports is essential for promoting physical and mental health, as the WHO advocates regular physical activity to prevent non-communicable diseases such as cardiovascular diseases, diabetes, and certain cancers. For autistic peoples, practice a physical activity is known to improve overall physical fitness, including parameters such as cardiovascular and muscular functions (9), as well as body composition and metabolic function (28). Regarding our results, we found that the mean of practice duration indicated was 115.22 ± 154.9 min per week. While this sport-dose is close to the WHO recommendation, that indicated that individuals must engage at least in 150 min of moderate-intensity physical activity per week, we noted a high level of variability. This result highlights a key factor, suggesting that the level of physical activity per week is insufficient for autistic individuals. This finding is consistent with existing literature which indicates a lower level of physical activity in autistic individuals compared to non-autistic individuals, especially among adults (21, 29). Interestingly, when we asked practitioners about the accessibility of sports practice for autistic people, nearly three-quarters of the respondents indicated a lack of accessibility, which may explain the difficulties in following the WHO recommendations. Here, the results revealed a high rate of sport participation, despite a commonly reported perception of inaccessibility, highlighting an apparent paradox. This discrepancy may suggest that those who do engage in sports are particularly motivated and willing to overcome the substantial barriers reported by non-participants. Their involvement could reflect a strong personal commitment, the presence of specific support systems, or the use of adaptive strategies that help them navigate challenges others may find prohibitive. Similarly, more than half of the non-participants indicated that they refrain from engaging in sports due to a lack of facilities adapted to the needs of autistic individuals, whether due to geographic or financial inaccessibility.

The sport participation rate observed in the present study (71.2%) is higher than those typically reported in the literature. For example, previous studies have found participation rates of 52.8% among Australian children at age 9% and 42% among Australian adults (30, 31). Interestingly, our findings are closer to those observed in the general adult population (71%, 31). On a broader international scale—particularly in studies conducted in North America—sport participation among autistic individuals tends to be even lower (32). While cultural and contextual differences between countries must be considered, it is also important to acknowledge that the recruitment method used in the present study, primarily via sport organizations, has increased the risk of sampling bias leading to an overestimation of sport participation rates among autistic respondents.

Beyond of the aim to improve physical fitness and physical and mental health, the use of sport with autistic individuals appears essential to positively modulate autism characteristics. Whether for the decrease of stereotypy (18, 33, 34), improvement of social skills (35–37), emotional regulation (13, 38) or cognitive functions (39–41), sport appears to be a relevant candidate for enhancing the quality of life of autistic people. However, the dose seems to be a crucial factor for benefiting from physical practice, as some studies have shown, for example, that 16 weeks, rather than 8, are necessary to significantly impact anxiety (42), or that increasing the frequency from 1 to 3 to 5 times per week significantly enhances the magnitude of observed changes (43). While no differences were observed between genders, it should be noted that the frequency of practice varied across age groups, being higher at older ages. This may not be specific to autism-related characteristics but rather linked to daily life organization, as older individuals tend to have more free time for leisure and hobbies. Additionally, the average session duration was also longer in older age groups. Similarly, this could be related to the general organization of sports practice, as session durations are typically shorter for children due to various factors, including attention span. Overall, while the literature on the chronic effects of sports interventions reports an average of 2.6 ± 1.4 sessions per week, with a mean session duration of 56.5 ± 19.3 min (11) and given the fact that mostly children are tested (more than 81% under the age of 12), the present study reveals that scientific literature does not systematically reflect current practice.

Thus, it seems essential to develop solutions to increase the accessibility of sports practice for autistic individual in order to align with general recommendations and fully leverage the benefits of physical activity.

### The type of practice: a key factor in the development of sports for autistic individuals

Thanks to the responses from sport practitioners, this study provides innovative insights into the types of practice favored and preferred by autistic individuals.

Firstly, our results showed that individual practices were predominant, accounting for nearly 80% of activities. This preference for individual practices raises an important point, which could be explained by interpersonal barriers such as lack of social support or differences in social skills (44–46). It worth mentioning that, and on the contrary to common belief, the existing literature probed that this type of activity still improves social skills and reduce communication deficit (33, 41, 47, 48). However, it appears that group-based practice tend to have a more pronounced impact on social behavior (11, 49), and that parents often perceive greater influence of group sports (50).

Secondly, a notable finding is the predominance of individual and aquatic sports among respondents, with over 20% reporting participation in swimming or other aquatic activities—placing it at the top of the list of practiced physical activities. This result is consistent with existing literature, which highlights the popularity of swimming among autistic individuals (11, 34). This preference may be explained by several characteristics of aquatic activities that align well with the sensory and cognitive profiles associated with autism. Swimming typically takes place in structured and predictable environments, which may help reduce anxiety. The sensory properties of water—such as pressure, temperature, and buoyancy—can be calming and regulating, offering a sense of weightlessness and controlled movement. Furthermore, aquatic sports generally involve fewer social demands and reduced interpersonal interactions, making them more accessible and enjoyable for individuals who may find group dynamics or team-based sports challenging.

Thirdly, we found that autistic people tend to prefer predictable activities, commonly referred to as open-skill sports, rather than unpredictable practices, known as closed-skill sports. This preference can undeniably be explained by the characteristics of autistic individuals, who often exhibit a strong inclination toward structured and predictable activities (11, 34) due to differences in sensory processing, executive functioning, and cognitive flexibility. Such activities provide a stable and controlled environment, reducing uncertainty and sensory overload, which might otherwise lead to stress and anxiety. Paradoxically, the very cognitive functions that make unpredictable activities challenging for autistic individuals are those that benefit the most from engaging in open-skill sports. It is well established that these sports, such as team sports and racket sports, enhance cognitive abilities more effectively than closed-skill sports (51–55). Their dynamic and unpredictable nature fosters continuous decision-making, attentional control, and cognitive flexibility, which are key areas of difficulty for autistic individuals but also potential targets for improvement through adapted practice. Thus, it would be beneficial to offer adapted open-skill sports to autistic individuals, ones that promote both physical and cognitive functions while minimizing anxiety.

Finally, another important point is that, according to findings, two-thirds of autistic individuals have reported engaging exclusively in recreational activities rather than competitive ones. This result raises a new question: is this a matter of personal preference, or are competitive activities simply less accessible to autistic individuals?

## Conclusions

The present study indicates that, quantitatively, autistic individuals tend to engage in less physical activity than recommended by global organizations such as the WHO or scientific literature. Qualitatively, their typical sports practice is predominantly non-competitive, individual, and focused on closed-skill activities, with aquatic activities being the most common. Contrary to common belief, while these activities provide a calm, safe, and predictable environment, they also contribute to the development of communication and social skills in autistic individuals.

However, the lower interest of autistic people in competitive, team-based, or open-skill activities (e.g., soccer, racket sports, or combat sports) may partly stem from misconceptions about the potential barriers they face in these contexts. The study reveals that the main obstacles to sports participation among autistic individuals remain limited accessibility and a lack of communication with their families and caregivers.

This study has several limitations that suggest avenues for future research. First, the survey was distributed through ERASMUS + project partners and in a limited number of languages, potentially restricting the sample to certain countries. More importantly, the autistic individuals reached through the project's communication channels, within networks promoting sports participation, may already be aware of and engaged in sports. This could lead to an underestimation of the proportion of non-practitioners and, consequently, limit insights into the reasons for non-participation. Future large-scale studies specifically targeting non-practitioners could help fill this gap. Also, it would also be of interest, with a larger and more balanced number of respondents per country, to conduct a country-specific analysis. This would help to account for disparities in socioeconomic development and autism-related infrastructure between countries. Another limitation concerns the heterogeneity of autism spectrum disorder. Stratifying the population based on specific characteristics, such as predominant social processing deficits, motor impairments, stereotyped and repetitive behaviors, or language and cognitive difficulties, would be highly relevant. Barriers to physical activity, as well as the types of sports practiced, are likely to differ depending on these characteristics. However, the sample size in our study did not allow such an analysis.

In conclusion, this study once again shows the importance of raising awareness about the benefits of sports for autistic individuals. It is essential to fight misconceptions and biases surrounding autism and sports, particularly among coaches, families, and caregivers. However, this study presents several limitations. First, a substantial part of the data relies on reports from proxies rather than directly from autistic individuals, which may introduce some discrepancies in the responses. Additionally, the use of self-reported data inherently carries the risk of subjective inaccuracies. The online survey format, while convenient for reaching a broad audience, may have led to a selection bias favoring participants interested in sports, and consecutively already engaged in sport practice. Also, given the well-known heterogeneity of autism in terms of symptoms and individual characteristics, it is difficult for a standardized questionnaire to fully reflect the specific needs and experiences of each respondent within this diverse population. The study did not specifically investigate which types of facilities were considered lacking, nor did it explore participants’ detailed perceptions of what accessibility entails in this context, thereby limiting the depth of understanding regarding environmental barriers and potential avenues for improvement.

Future research should include qualitative studies, based for instance on structured interviews, to explore in greater depth the barriers and facilitators to sports participation from the perspective of autistic individuals themselves, providing a more nuanced understanding of their lived experiences. Intervention studies are also needed to test the effectiveness of adapted sports programs and identify which approaches are most supportive. In addition, research should focus on the specific features of sports that yield the greatest benefits for different autistic individuals, recognizing the diversity within the spectrum. Longitudinal studies would be valuable to track changes in participation and outcomes over time, while cross-cultural comparisons could highlight on how social, cultural, and systemic factors influence access and engagement in sports across different contexts.

## Data Availability

The raw data supporting the conclusions of this article will be made available by the authors, without undue reservation.
